# Exploring boundary conditions of physical activity maintenance: A secondary analysis of time-series data from a weight-loss intervention

**DOI:** 10.1080/21642850.2025.2554980

**Published:** 2025-09-11

**Authors:** Dario Baretta, Guillaume Chevance, Shadia J. Mansour-Assi, Victoria Lawhun Costello, David Wing, Eric B. Hekler, Jennifer Inauen, Job Godino, Claudio R. Nigg

**Affiliations:** a Institute of Psychology, University of Bern, Bern, Switzerland; b Inserm, EHESP, Irset, University of Rennes, Rennes, France; c Barcelona Institute for Global Health, Barcelona, Spain; d Center for Wireless and Population Health Systems, UC San Diego, La Jolla, CA, USA; e Laura Rodriguez Research Institute, Family Health Centers of San Diego, San Diego, CA, USA; f Institute of Sport Science, University of Bern, Bern, Switzerland

**Keywords:** Physical activity maintenance, moderate-to-vigorous physical activity, boundary conditions, phenomena-driven research, temporal trajectories

## Abstract

**Objective:**

A key concept in health psychology is behavioral maintenance. However, previous research has struggled to establish shared conceptualizations and operational definitions. This study aimed to contribute to this debate by examining whether a simple conceptual proposition of physical activity maintenance as ‘the performance of physical activity according to an intended target threshold over a specific period of observation’ can be empirically supported, and under which boundary conditions. Specifically, we explored different formulations of two boundary conditions: activity threshold and timescale of change.

**Methods:**

We analyzed 350 time series (length = 182 days) of moderate-to-vigorous physical activity (MVPA) collected daily with Fitbit from participants in a weight loss intervention. All participants reported an intention to engage in at least 150 min of MVPA per week over the following six months. Activity thresholds were defined based on each participant’s baseline MVPA. Generalized Additive Models were used to model individual trajectories across varying timescales (7, 14, 28, and 56 days).

**Results:**

At short timescales (7–14 days) trajectories crossed the threshold frequently, indicating high variability. At longer timescales (28–56 days) trajectories were more stable, with participants tending to stay either above or below their threshold, aligning with our target conceptualization of maintenance. Relaxing the threshold by 10–20% relatively increased the proportion of participants classified as maintainers, though maintenance remained uncommon for participants with higher thresholds.

**Conclusions:**

Our findings provide initial evidence on which boundary conditions support detecting physical activity maintenance as conceptually defined. These results underscore the importance of systematically testing boundary conditions to advance understanding of behavioral maintenance.

**Trial registration:** ClinicalTrials.gov identifier: NCT03907462.

## Introduction

Regular physical activity is associated with reduced risks of mortality caused by non-communicable diseases (Rhodes et al., 2017; Warburton & Bredin, 2017). Many of the benefits and long-term health effects are achieved when physical activity is consistently performed over time (i.e. years) (Reiner et al., 2013; Shortreed et al., 2013), which is therefore a major goal for physical activity interventions (Murray et al., 2017). The broad concept of long-term and sustained physical activity is known in the literature as ‘physical activity maintenance’ (Nigg et al., 2008). Despite an intuitive understanding of what maintenance means, this research topic is characterized by conceptual arguments and confusion, and discordant operationalization resulting from a wide array of experiences, ideas, conventions, and notions that have formed over time within the field of health psychology (Dunton et al., 2022; Kahlert, 2015; Rhodes & Sui, 2021).

A valuable approach to advancing this debate and improving clarity around the concept of physical activity maintenance and its operationalization is through epistemic iteration. According to it, the essential components of scientific inquiry, such as concept definition, phenomena description, measurement, statistical analysis, and theorizing, are refined in repeated cycles, with progress in any one component feeding back to improve the others (Bringmann et al., 2022). For instance, recent improvements in measuring physical activity with activity trackers have made it possible the collection of intensive longitudinal data, which improves our ability to develop both theoretical and conceptual insights into physical activity maintenance, as well as testing alternative operationalizations (Dunton et al., 2021). In epistemic iteration, it is important that researchers do not become fixed on a single component of the cycle (e.g. theorizing or measurement); instead, they should work through the entire iterative process and revisit the target concept as necessary to ensure its clarity and progression (Bringmann et al., 2022).

We argue that recent research on physical activity maintenance has primarily focused on conceptual definitions and theorizing (Dunton et al., 2022; Kwasnicka et al., 2016; Rhodes & Sui, 2021), in part overlooking other components of epistemic iteration, such as phenomena^1^ description. The aim of this paper is to complement and build upon previous conceptual work, focusing on the description of phenomena and their boundary conditions (Busse et al., 2017; Haig, 2013). Specifically, we aim to examine whether certain conceptual propositions of physical activity maintenance can be empirically supported by the data, and under what operational conditions.

As each component of the epistemic iteration builds upon existing understandings, a working definition of physical activity maintenance is necessary to serve as a conceptual target for our phenomenon-driven research. We adopted a pragmatic and parsimonious approach, extracting a simple proposition from previous conceptualizations (Dunton et al., 2022; Kahlert, 2015; Murray et al., 2017; Rhodes & Sui, 2021; Seymour et al., 2010), to capture the major and partly agreed upon aspects of physical activity maintenance. Specifically, we intend physical activity maintenance as ‘the performance of physical activity according to an intended target threshold over a specific period of observation’. This conceptualization does not claim to be definitive or superior to others, but we consider it sufficient for the purpose of this study. By shifting the focus to the phenomena description component within the epistemic iteration framework, we aim to empirically assess whether this conceptual definition is consistently supported by observed data patterns (i.e. the phenomena) and under which boundary conditions.

### Boundary conditions of physical activity maintenance

A key strength of the phenomena-driven approach is that it allows us to assess under which boundary conditions a given theoretical concept is empirically supported. Boundary conditions refer to the ‘limitations on the propositions generated from a theoretical model’ (Whetten, 1989, p. 482). They clarify the conditions under which a theoretical concept or proposition holds true. Identifying boundary conditions is essential for establishing stable descriptions of a system's behaviors and generating descriptions of phenomena, ultimately playing a critical role in the development and testing of scientific theories (Bursten, 2021; Busse et al., 2017; Whetten, 1989).

In the context of physical activity maintenance, boundary conditions may refer to the values of certain key operational definitions that can contribute to shape how the concept of maintenance is measured and interpreted. Recently, Dunton and colleagues (2022) provided a list of various operational definitions. In this study, we examined how different formulations of two boundary conditions, *timescale of change* and *activity threshold*, among other possible factors, impact our ability to observe a phenomenon that aligns with the target conceptualization of maintenance introduced above.

#### First boundary condition: timescale of change

The way researchers treat temporal aspects (e.g. time aggregations, duration of steady states) can profoundly affect observability of empirical phenomena, with important implications for theory development and concept refinement (George & Jones, 2000; Langener et al., 2024; Scholz, 2019). A critical prerequisite for investigating time-related boundary conditions, such as the timescale of change, is to access high-resolution time series data (Chevance, Perski, et al., 2021). This type of intensive longitudinal data is essential to explore the temporal dynamics and trajectories of physical activity behavior and its maintenance (Dunton et al., 2021).

**Figure 1.**
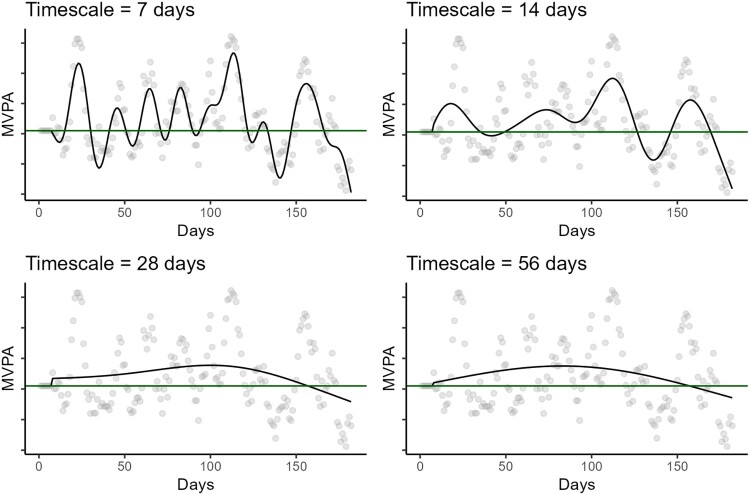
Physical activity trajectories modeled with increasingly larger time parameters. Note. Black lines depict participants’ physical-activity trajectories; green horizontal lines mark the activity threshold. Each panel shows the same time series modeled with progressively longer timescale parameters. When a short timescale is used (e.g. 7 days, top left), the trajectory crosses the threshold multiple times. As the timescale parameter increases, crossings become less frequent.

Research using intensive longitudinal data has shown that physical activity is a highly volatile behavior (Shang et al., 2018), characterized by intra-individual fluctuations not only over days (Baretta et al., 2023; Chevance, Baretta, Heino, et al., 2021) but also within the same day (Nigg et al., 2022; ten Broeke et al., 2020). An effective approach to explore such fluctuations and their timescale is to analyze the trajectory underlying physical activity time series using statistical approaches capable of modeling non-linear temporal patterns (e.g. Baretta et al., 2023, 2025). When conducting this type of analysis, the selection of model parameters that define the timescale of the fluctuations holds substantial implications for the description of phenomena. By varying these model parameters, it is possible to examine change processes at different timescales (Spruijt-Metz et al., 2015). For example, models can be specified to capture fluctuations occurring at shorter (e.g. weekly) or longer (e.g. monthly) timescales, with implications for determining whether the trajectory is above or below the maintenance threshold (see Figure 1). Despite this relevance, no empirical work has yet tested how choosing different timescales impact conclusions about physical activity maintenance (Dunton et al., 2021).

#### Second boundary condition: physical activity threshold

A further key operational definition of physical activity maintenance is the activity threshold, which refers to the amount of physical activity (e.g. volume expressed in minutes per week) that represents the target level for behavior change and maintenance (Dunton et al., 2022). In physical activity, thresholds might correspond, among the others, to a ‘clinical’ standard for reducing the risk of non-communicable diseases (e.g. 150 min of MVPA/week) (Ding et al., 2020) or specific intervention goals (Murray et al., 2017).

Activity thresholds may be challenging to achieve and maintain depending on their intrinsic difficulty (e.g. increasing the number of daily steps by a factor of two is more demanding than by a factor of 1.5; Chevance, Baretta, Golaszewski, et al., 2021). Additionally, thresholds may have different effects depending on whether individuals need to first initiate behavior change to reach them, or whether their behavior already meets the threshold and must ‘only’ be maintained. All these possible factors position the activity threshold and its relation to individual characteristics (e.g. stage of change, intention) as a critical boundary condition to consider when conceptualizing physical activity maintenance. Examining different formulations of activity thresholds allows us to assess how those choices influence the observability of phenomena that align with the conceptualization of maintenance.

### Aim of the study

The aim of this study was to examine whether different formulations of two boundary conditions, *timescale of change* and *activity threshold*, impact our ability to observe a phenomenon consistent with the conceptualization of physical activity maintenance as the performance of physical activity according to an intended target threshold over a defined observation period. Following a previous meta-analysis (Murray et al., 2017), we set the observation period at six months. More specifically, we aimed at quantifying whether and how often person-specific physical activity trajectories crossed the activity threshold, with more frequent transitions below the threshold indicating a phenomenon that diverges from the proposed conceptualization.

For the first boundary condition, we systematically varied the model parameters that determine the timescale of change to capture potential fluctuations occurring over 7-, 14-, 28-, and 56-day intervals. These values represent successive doublings of seven days, the standard timeframe for health-related physical activity targets, particularly when expressed in MVPA (Bull et al., 2020). This multiplicative progression keeps each timescale easy to interpret in both days and whole weeks. As each timescale places an upper limit on the fluctuations detectable within the six-month observation window, these limits must be considered when interpreting the results, as they determine the model’s sensitivity to short- versus long-term changes in behavior.

For the second boundary condition, we examined different activity thresholds based on participants’ baseline MVPA, creating two groups. The first group, named BL-Inactive, included those performing fewer than 150 min per week at baseline who aimed to reach and maintain at least this threshold during the same period. The second group, named BL-Active, comprised those already achieving more than 150 min of MVPA per week at baseline and intending to maintain that level over the next six months.

## Methods

We used data from the SMART 2.0 study, a 24-month (96 weeks), three-arm, parallel-group randomized controlled trial targeting weight loss among young adults with overweight or obesity (Mansour-Assi et al., 2022; ClinicalTrials.gov Identifier: NCT03907462). The trial was conducted between April 2019 and February 2024, in San Diego, USA. All relevant ethical regulations have been followed, and the study was approved by the Human Research Protections Programs at University of California, San Diego (protocol #181862).

After providing informed consent, the participants were stratified by sex and university/college affiliation and were randomized within each stratum at a 1:1:1 ratio to one of three groups: (1) treatment one, (2) treatment two, or (3) a control group. Group assignment remains unknown for the present analyses. Participants in each group received a consumer-level wearable activity tracker and connected scale from Fitbit, which includes access to the Fitbit smartphone and web-based application ecosystem where they were able to track their MVPA and the MVPA goal. Participants in the control group received no instruction to change the default Fitbit goal (150 min MVPA/week). Participants in the two treatment conditions were given the goal to achieve 225 min/week of MVPA, starting from the second week. Further details on the intervention content are described in the trial protocol (Mansour-Assi et al., 2022). For the current study, we examined physical activity trajectories considering the first 182 days (i.e. 6 months) of data. We chose this length because it reflects participants’ intentions of being active according to a specific goal as assessed at baseline (see next section).

### Participants

Participants (N = 638) were young adults (aged 18–35 years) recruited from three universities and five community colleges in San Diego, USA. The inclusion criteria include, (1) overweight or obese (25 ≤ BMI < 40 kg/m2); (2) available for a 24-month intervention, (3) affiliated with one of the target universities as a student, staff, or alumni, (4) willing and able to use social media, a smartphone, text messaging, and Fitbit devices and app, and (5) willing and able to engage in MVPA. The exclusion criteria include, (1) comorbidities of obesity or conditions that prohibit compliance with the study protocol, (2) a recent cardiovascular event, (3) currently being treated for malignancy and/or an eating disorder, (4) planning to have weight loss surgery or enroll in a weight loss program, (5) loss of more than 15 pounds within the past 3 months, and (6) pregnancy or planning pregnancy within 24 months.

For the purpose of the current study, we also excluded from the analysis those participants who (i) did not intend to be active at a moderate intensity for at least 150 min per week over the next 6 months, or (ii) had more than 20% missing daily observations, according to statistical recommendations and previous research on physical activity time series (Baretta et al., 2023; Bolger & Laurenceau, 2013; Chevance, Baretta, Heino, et al., 2021). The two analytical groups described above were then defined based on their baseline activity level (see Supplementary Figure 1). The first analytical group, ‘BL-Inactive’ (n = 50), encompassed participants who, at baseline, engaged in less than 150 min of MVPA per week. For this group, the target physical activity threshold, against which the trajectory was compared, was set at 150 min of MVPA per week. The second analytical group, ‘BL-Active’ (n = 300), included participants who, at baseline, engaged in more than 150 min of MVPA per week. For this group, the target physical activity threshold, corresponded to the person-specific baseline MVPA value (see the next section about measures). To note, the threshold for subsample ‘BL-Inactive’ is lower than the threshold for subsample ‘BL-Active’ as the baseline MVPA value of participants in the second subsample already exceeded 150 MVPA/week.

### Measures

#### Baseline self-report measures

At baseline participants self-reported their age and sex while weight and height were measured objectively in the lab and used to compute participant’s BMI in kg/m^2^. Intention to be active at a moderate intensity for at least 150 min a week over the next 6 months was measured on a 5-point Likert scale ranging from 1 (Strongly disagree) to 5 (Strongly agree). To be included in the analytic sample, participants had to report a score of 4 or 5 on this item.

#### Physical activity

MVPA was continuously collected through a wrist-worn activity tracker from Fitbit (i.e. Fitbit Charge; Fitbit Inc, San Francisco, CA, USA) regardless of the duration of physical activity bouts (Jakicic et al., 2019). Participants were asked to wear the device continuously according to manufacturer recommendations (i.e. to wear the device on one’s non-dominant arm and up to three finger widths above the wrist bone). Fitbit classifies MVPA as any activity equal or larger than 3.0 metabolic equivalents (METs), a threshold consistent with standard definitions in the literature (Haskell et al., 2007). MVPA was preferred over other physical activity indicators (e.g. step-count) as the intention to engage in MVPA was an inclusion criterion and reflects the participants’ goal at baseline. Additionally, although MVPA is not a direct measure of specific movement patterns, it harmonizes diverse types of physical activity into a single, comparable metric and represents the basis of major public-health guidelines (Bull et al., 2020).

We identified invalid days in case (1) no data was collected by the Fitbit on a specific day, or (2) the number of sedentary minutes was equal to 1440 per day (i.e. 24 h), which represents an unrealistic value. Invalid days were subsequently recoded as missing. Previous validation studies of Fitbit Charge devices showed a tendency to overestimate MVPA (Matlary et al., 2022). Even though this aspect is problematic when the Fitbit wristband is used in a setting that requires strong criterion validity (e.g. a clinical trial in which Fitbit-assessed MVPA represents the clinical outcome), it represents a minor threat here since the tendency to overestimate is systematic and not hypothesized to influence the evaluation of MVPA patterns of change over time.

##### Baseline physical activity

Baseline physical activity was calculated for each participant by summing the number of minutes of MVPA during the first week of Fitbit data collection. We chose one week as participants in the treatment arms received their weekly physical activity goals one week after the start of Fitbit data collection, which could have influenced their activity levels. To examine this assumption, we compared baseline estimates derived from one versus two weeks of data. The two-week baseline yielded slightly lower MVPA estimates in the active group (M₁ = 456 min, M₂ = 437 min; *t* = 1.02, *p* = .309) but substantially higher estimates in the inactive group (M₁ = 84 min, M₂ = 126 min; *t* = −3.07, *p* = .003). Because such higher baseline values with the two-week approach may reflect an intervention effect in less active individuals, we retained the one-week period as the most appropriate and conservative definition of baseline for this study.

### Data analysis

#### Time series characteristics

The length of the time series was set to 6 months (182 days) for all participants. The median number of missing days per participant was 2 (range = 0 - 34 days) in the BL-Inactive group, and 4 days (range = 0 - 36 days) in BL-Active one. Missing days were imputed using the Kalman Filter method which is recommended for univariate time series (Gómez & Maravall, 1994; Moritz & Bartz-Beielstein, 2017).

#### Analytical approach

We compared each participant’s MVPA trajectory against the physical activity threshold specific to their group. To do this, we adopted an idiographic (person-specific) approach and applied a statistical method previously used to identify trajectories from step count time series (Baretta et al., 2023; Baretta, Koch, et al., 2025). Notably, we fitted Generalized Additive Models (GAMs) with the *gam* function from the *mgcv* package in R (Wood, 2017) to model each participant’s MVPA over the six-month observation period. GAMs allow to fit smoothed functions as combinations of multiple low-level functions (e.g. linear function, quadratic function, logarithmic function), making them well suited for non-linear trends. In our models, weekly MVPA was first calculated on a seven-day rolling sum, then regressed on study day. For each participant, we then subtracted the relevant activity threshold value (e.g. 150 min MVPA week for those included in the BL-Inactive group) from the fitted MVPA trajectory. The resulting difference was recoded as a binary outcome indicating whether, on each day, the participant’s MVPA trajectory was above or below the threshold. To make this comparison at different timescale of change (7, 14, 28, 56 days), we repeated this analytical procedure four times. Each time, we adjusted the *k* parameter in the *gam* function, which controls the number of basis functions and thus the model’s flexibility in capturing non-linear trends. We set the *k* parameters by dividing the six-month observation period by each timescale, permitting one slope (i.e. fluctuation) every 7, 14, 28, and 56 days, respectively.

Finally, we aggregated individual statistics to quantify how often, on average, person-specific trajectories crossed their target thresholds under each combination of timescale and activity threshold. Given that the maintenance threshold in the BL-Active group was not fixed but it corresponded to the person-specific baseline MVPA, in this group we also examined the association between baseline MVPA and (i) total transitions across the threshold, (ii) total days spent below the threshold, and (iii) ending the six-month period below the threshold.

#### Multiverse analysis

We applied a multiverse approach (Steegen et al., 2016) to examine further alternative operationalizations of the maintenance threshold. We re-ran all analyses using 90% and 80% of the original threshold in both groups, effectively counting only transitions that fell at least 10% or 20% below the original threshold. For example, if the original threshold was 200 min MVPA/week, the 90% threshold corresponded to 180 min MVPA/week. Additionally, within the BL-Active group, we repeated the analyses including also the 150-minute threshold as it represents the common, minimum target threshold across all intervention arms.

## Results

### Participants characteristics

Baseline characteristics for the analytical sample (BL-Inactive and BL-Active) appear in Table 1. Compared with excluded participants, the analytical sample was slightly older and reported stronger intentions to increase MVPA. Over the six-month study period, the BL-Active group averaged 375 min of MVPA per week (range 0–2,315 min; median 327 min); the BL-Inactive group averaged 175 min of MVPA per week (range 0–1,532 min; median 140 min).

**Table 1.** Sociodemographic characteristics, BMI and physical activity at baseline.

	BL-Inactive (N = 50)	BL-Active (N = 300)	Overall (N = 350)	Excluded sample (N = 288)	Group differences (*p*-value)^a^
*Sex*					0.303
Male	14 (28%)	135 (45%)	149 (43%)	111 (39%)	
Female	36 (72%)	165 (55%)	201 (57%)	177 (61%)	
*Age*					<0.001
Mean (SD)	25.7 (4.6)	23.3 (4.7)	23.6 (4.8)	22.2 (4.0)	
Min. Max	18, 35	18, 35	18, 35	18, 35	
*BMI (kg/m2)*					0.767
Mean (SD)	30.0 (4.0)	30.0 (3.5)	30.0 (3.6)	29.9 (3.7)	
Min. Max	25.1, 39.9	25.1, 39.9	25.1, 39.9	25.1, 39.7	
*Intention to do 150 mins MVPA*^b^					0.014
Mean (SD)	4.42 (0.50)	4.60 (0.49)	4.58 (0.49)	4.35 (0.89)	
Min. Max	4.00, 5.00	4.00, 5.00	4.00, 5.00	1.00, 5.00	
*Baseline MVPA (1st week of Fitbit data)*					–
Mean (SD)	84 (42)	456 (241)	403 (259)	–	
Min. Max	0, 149	155, 1,366	0, 1,366	–	

Note***.*** ^a^Group difference are between the included (BL-Inactive + BL-Active, column ‘Overall’) and excluded sample. Wilcoxon rank sum test was used for group differences in continuous variables while Fisher's exact test used for group differences in categorical variables; ^b^Intention to be active at a moderate intensity for at least 150 min a week over the next 6 months. Baseline MVPA data based on Fitbit was not reported for the excluded sample due to missing values, which could bias the MVPA estimate.

### Impact of boundary conditions on the observability of maintenance phenomena

#### BL-active group

**Figure 2.**
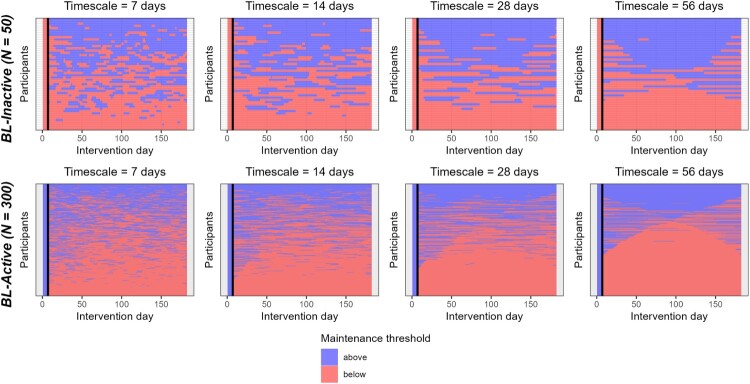
Histograms and heatmaps showing phase transitions across the maintenance thresholds in the two groups. Note. The Y axis represents a unique participant, while x axis indicates progressive days since the intervention started. The color scale distinguishes whether the person specific MVPA trajectory is above (blue) or below (red) the target thresholds. Vertical black lines indicate the first week of Fitbit data used to calculate baseline MVPA.

The number of transitions across the maintenance threshold decreased as the timescale of change increased (Table 2, Figure S2). At the stricter maintenance thresholds (baseline MVPA and its 90%), participants were more likely to be below the threshold at the end of the study period, whereas at the 150-min threshold they were more likely to be above it (Chi-squared tests, Table 2). Heat-map inspection (Figure 2; Supplementary Figures S4-S5) confirms this pattern: as the timescale lengthens, MVPA trajectories show fewer crossings of the threshold, and the more lenient the threshold, the more participants end the observation period above it.

**Table 2.** Frequency of participants crossing different activity thresholds based on their group.

	Time scale of change			
	7 days	14 days	28 days	56 days
*BL-Active*				
*Target threshold (Baseline MVPA)*				
Mean (SD)	6.47 (3.73)	3.31 (2.05)	1.97 (1.33)	1.20 (0.71)
Min, Max	0, 17	0, 9	0, 6	0, 3
Participants with final MVPA below threshold^a^	208 (69%)***	198 (66%)***	201 (67%)***	203 (68%)***
*90% target threshold*				
Mean (SD)	6.58 (3.45)	3.34 (1.98)	1.86 (1.26)	1.13 (0.75)
Min, Max	0, 15	0, 9	0, 5	0, 3
Participants with final MVPA below threshold^a^	194 (65%)***	184 (61%)***	185 (62%)***	180 (60%)***
*80% target threshold*				
Mean (SD)	6.58 (3.39)	3.26 (2.07)	1.79 (1.24)	1.02 (0.81)
Min, Max	0, 15	0, 9	0, 5	0, 3
Participants with final MVPA below threshold^a^	177 (59%)**	173 (58%)**	170 (57%)*	150 (50%)
*150 mins MVPA*				
Mean (SD)	4.01 (3.59)	1.68 (1.91)	0.80 (1.18)	0.31 (0.63)
Min, Max	0, 15	0, 9	0, 5	0, 3
Participants with final MVPA below threshold^a^	79 (26%)***	81 (27%)***	67 (22%)***	45 (15%)***
*BL-Inactive*^b^				
*Target threshold (150 mins MVPA)*				
Mean (SD)	6.19 (3.85)	2.75 (2.02)	1.07 (1.03)	0.54 (0.65)
Min, Max	0, 13	0, 9	0, 4	0, 2
Participants with final MVPA below threshold^a^	27 (54%)	31 (62%)	27 (54%)	27 (54%)
*90% target threshold*				
Mean (SD)	6.10 (4.02)	2.82 (1.92)	1.15 (1.09)	0.53 (0.60)
Min, Max	0, 14	0, 9	0, 4	0, 2
Participants with final MVPA below threshold^a^	27 (54%)	26 (52%)	24 (48%)	28 (56%)
80% target threshold				
Mean (SD)	6.00 (3.87)	2.52 (1.86)	1.21 (1.14)	0.51 (0.61)
Min, Max	0, 14	0, 6	0, 4	0, 2
Participants with final MVPA below threshold^a^	30 (60%)	24 (48%)	27 (54%)	28 (56%)

Note. ^a^Indicates the number of participants whose trajectory fell below the activity threshold at the end of the 6-month observation period. A chi-squared test was used to assess whether significantly more participants ended above or below the threshold. Significance levels: *p < .05, **p < .01, **p *<* *.001*. ^b^The first transition in the BL-Inactive group was excluded, as it reflects behavioral adoption, that is, the initial attainment of the target threshold.

Table 3 reports correlations between each participant’s baseline MVPA and (i) total transitions, (ii) total days spent below the threshold, and (iii) ending the six-month period below the threshold. Results are shown for the main threshold (baseline MVPA) and for the 90%, 80%, and 150-min variants. At the 7- and 14-day timescales, higher baseline MVPA correlated with fewer total transitions (ρ = −0.354, *p* < 0.001 and ρ = −0.247, *p* < 0.001) when the target threshold was the baseline MVPA itself. This association became weaker when using a more relaxed 90% threshold (ρ = −0.200, *p* < 0.001 at the 7-day timescale) and disappeared when moving to the 80% threshold. At this threshold, however, we observed a positive association between baseline MVPA and number of transitions at longer timescales (ρ = 0.152, *p* < 0.01 and ρ = 0.195, *p* < 0.001). When using the 150-minute MVPA threshold, we observed a negative association across all timescales.

**Table 3.** Correlation between baseline MVPA and number of transitions, having final MVPA below threshold, and days spent below the threshold.

	Timescale of change			
	7 days	14 days	28 days	56 days
*Correlation between baseline MVPA and number of transitions*				
Target threshold	−0.354***	−0.247***	−0.124*	0.105
90% target threshold	−0.200***	−0.089	−0.013	0.177**
80% target threshold	−0.067	0.063	0.152**	0.195***
150 mins MVPA	−0.451***	−0.391***	−0.325***	−0.318***
*Correlation between baseline MVPA and having final MVPA below threshold*				
Target threshold	0.290***	0.319***	0.392***	0.296***
90% target threshold	0.239***	0.285***	0.374***	0.280***
80% target threshold	0.228***	0.255***	0.330***	0.272***
150 mins MVPA	−0.208***	−0.188**	−0.131*	−0.236***
*Correlation between baseline MVPA and days spent below the threshold (trajectory-based)*				
Target threshold	0.570***	0.568***	0.570***	0.547***
90% target threshold	0.535***	0.540***	0.552***	0.511***
80% target threshold	0.480***	0.492***	0.484***	0.445***
150 mins MVPA	−0.459***	−0.426***	−0.339***	−0.325***

Note. Coefficients are Spearman’s ρ; * *p* < .05; ** *p* < .01, *** *p* < .001. Trajectory-based means that days spent below the threshold were inferred using the fitted values from the GAMs.

Additionally, Table 3 shows that, using each participant’s own baseline as the target threshold, higher baseline levels were associated, across all timescales, with a greater probability of ending the six-month period below the threshold. This association consistently decreased when the thresholds were lowered to 90% and 80% of baseline but remained significant. At the 150-minute MVPA threshold, the correlation reversed, indicating a stronger association between higher baseline MVPA and a greater probability of ending the six-month period above the threshold. Similarly, we observed that at stricter thresholds (baseline or its 90% and 80%), higher baseline levels were associated with more days spent below the threshold, across all timescales. At the 150-minute threshold, the correlation again reversed, showing a stronger association between baseline MVPA and fewer days spent below the threshold.

Supplementary Table S1 and Figure S2 summarize, for each combination of timescale and threshold, how many participants had an MVPA trajectory that aligns with the current conceptual definition of behavioral maintenance. Lowering the maintenance threshold from baseline MVPA to 90% and 80% did not change the proportion of participants whose trajectories fluctuated around the threshold, regardless of timescale. Conversely, when the threshold was lowered, the number of participants who maintained throughout increased, while the number who fell below the threshold and never returned decreased. Even so, at these reduced thresholds, the proportion who maintained throughout (i.e. had zero transitions across the threshold) remained modest, ranging from 0.3–2.0% at the 7-day timescale to 11.7–28.0% at the 56-day timescale. At the lowest 150-minute threshold, the proportion of participants who maintained throughout rose from 22.7% at the 7-day timescale to 77.7% at 56 days.

#### BL-inactive group

In the BL-Inactive group, the number of phase transitions also declined as the timescale increased (Table 2, Supplementary Figures S3). Chi-squared tests (Table 2) revealed no significant tendency for participants to end the six-month period above or below any of these thresholds, irrespective of timescale. Figure 2 and Supplementary Figure S6 show this consistent trend: at longer timescales, the MVPA trajectories stabilize and converge into distinctive patterns with fewer fluctuations across the threshold.

Supplementary Table S1 and Figure S3 show that the proportion of participants who never reached the threshold (i.e. zero transitions) was relatively low, ranging from 4% at the 7-day timescale to 24–26% at 56 days. Those who reached the threshold and then maintained it ranged from 6–10% at the 7-day timescale to 28% at 28 days and to 40% at 56 days. At the 28- and 56-day timescales, lowering the threshold from 150 min MVPA to 90% or 80% of that value did not change this maintenance proportion, which remained at 28% and 40%, respectively across thresholds.

## Discussion

The current results provided insights into whether different formulations of two boundary conditions, timescale of change and activity threshold, affect the ability to detect data patterns consistent with the conceptualization of physical activity maintenance as the performance of physical activity according to an intended target threshold over a defined observation period.

Findings revealed that varying the timescale of change gives rise to different phenomena. These ranged from a highly dynamic scenario marked by repeated fluctuations to a scenario characterized by stable physical activity trajectories. At a shorter timescale (7–14 days), the results align with previous literature, suggesting that physical activity is a volatile behavior, often fluctuating around a target threshold over time (Chevance, Baretta, Heino, et al., 2021; Shang et al., 2018). These frequent crossings of the threshold suggest that, at short timescales, individuals often dip below the intended level, which does not conform to the conceptual definition of physical activity maintenance adopted in this study.

In contrast, at longer timescales of 28 or 56 days, the data revealed more stable trajectories, which aligns better with the target conceptualization of maintenance. Most of the individuals tended to either consistently remain above the threshold or fall below it and remain there. This polarization between two distinct behavioral patterns was observed both in the BL-Inactive group, who needed to initiate and then maintain behavior change, and in the BL-Active group, who had higher thresholds but did not need to initiate change. These more stable patterns were also present when relaxed thresholds (e.g. 90% or 80% of the original target) were applied, although they became especially pronounced when using a substantially lower threshold such as 150 min of MVPA per week in the BL-Active group. Here, most trajectories stayed above the threshold, indicating that 150 min, which is less than half of the group’s average baseline MVPA, was a comparatively easy level for them to sustain.

These findings highlight the importance of understanding the functional implications of short-term behavioral variability for long-term behavioral maintenance (Scholz, 2019; Spruijt-Metz et al., 2015). This understanding is essential for developing effective interventions and gaining a deeper insight into how behavior evolves over time. Modeling fluctuations in physical activity at shorter timescales offers valuable information about short-term behavioral variability (e.g. lapses), which could be useful for designing just-in-time adaptive interventions (Nahum-Shani et al., 2018). However, shorter fluctuations below the threshold do not necessarily result in a longer-lasting trend below it. For instance, we observed several instances of short-term lapses that ‘disappeared’ when analyzed using a longer timescale (see Figure 2, and Supplementary Figures S4-S6). In such cases, where the broader trend shows that the person generally meets the target threshold, it is important to question whether addressing every short-term lapse is necessary or beneficial. Future research should aim to develop approaches that can distinguish and predict whether short-term fluctuations are merely temporary or are early indicators of a long-term shift below the threshold.

Important insights come from the analysis conducted within the BL-Active group, where we compared each person’s baseline MVPA, used as their individual activity threshold, with the total number of transitions, the total number of days spent below the threshold, and the likelihood of ending the six-month period below the threshold. Across all timescales, the pattern was consistent: the higher a person’s initial activity level, the harder it was to stay above that level (more days were spent below the threshold), physical activity fluctuated less (fewer transitions), and the probability of ending below the threshold increased. These findings suggest that, for very active individuals, the baseline value, as quantified in this study, may be too demanding to maintain. More relaxed constraints, such as lowering the threshold by at least 20%, might represent a suitable strategy to purposefully overlook less pronounced lapses in favor of observing more stable trajectories (Supplementary Figure S4). Nonetheless, it is important to find the right balance, as lowering the threshold too much, for example, using the 150-minute MVPA threshold for this already active group, may set too easy a target. This would result in a scenario consistently characterized by fewer transitions, more days above the threshold, and a higher probability of ending the six-month observation period above it, regardless of timescale.

### Strengths and limitations

A key strength of this study is its use of the epistemic-iteration framework to test whether the target conceptualization of physical-activity maintenance is supported by empirical phenomena across different boundary conditions. To the best of our knowledge, this is the first study in the behavioral maintenance literature to make such a systematic, dialectical comparison between conceptual definitions and observed phenomena under varying operational choices. Additionally, this approach was made possible thanks to the use of intensive longitudinal data, which are necessary to investigate phenomena that unfold over time and are therefore integral for research on behavioral maintenance (Chevance, Perski, et al., 2021; Dunton et al., 2021). Another strength of the study lies in the use of GAMs for modeling physical activity trajectories, as they offer a flexible method for capturing smooth, gradual changes over time. Nonetheless, alternative approaches could also be employed with such data. For instance, change-point detection methods like regression trees may be more appropriate for modeling abrupt, rather than gradual, shifts in physical activity trajectories (Baretta, Mazéas, et al., 2025; Chevance, Baretta, Heino, et al., 2021). Methods capable of modeling both gradual and sudden changes have also been proposed in recent years (Albers & Bringmann, 2020; Smit, 2020); however, they tend to be more computationally demanding, and their application has thus far been limited to self-report data on Likert scales.

While this study offers valuable insights, it also has several limitations. First, we analyzed physical-activity time series from a sample of educated participants enrolled in a weight-loss intervention, which may limit the generalizability of our findings to observational settings or to interventions with different aims, content, or target population. Second, because participants’ assignment to specific trial arms was unavailable, we could not include group allocation in our analyses. However, this information was not critical to the present study, as our goal was to examine the maintenance phenomenon itself (i.e. to identify patterns of maintenance success and failure), rather than to evaluate the intervention’s effects. Third, our baseline assessment of physical activity was based on the first week of Fitbit data following study enrollment. We preferred this approach over using two weeks because participants in the treatment arms began receiving intervention content during the second week. However, it is possible that the act of enrolling in the study and beginning activity monitoring may itself have prompted participants to increase their activity in that initial week, potentially inflating baseline estimates. Fourth, prior research has revealed a tendency for Fitbit Charge devices to overestimate MVPA (Matlary et al., 2022). This overestimation could pose challenges in settings requiring high criterion validity, such as clinical trials where Fitbit-assessed MVPA serves as a direct clinical outcome. However, in our context, this issue represents a minor concern as the systematic nature of this overestimation did not hinder our examination of the boundary condition of timescale. At most, it may have led to an overestimation of the total number of phases during which participants were active above the threshold. Fifth, in the parent trial, participants’ intentions to engaged in at least 150 min of MVPA per week was assessed at baseline and during the 6, 12, 18, and 24-month follow-up assessments, rather than using more frequent measurements. As a result, we were unable to evaluate whether changes in physical activity during the first 6 months corresponded to changes in participants’ intentions. Last, as we analyzed physical activity trajectories using time series data spanning the first 6 months of the intervention, it is possible that we did not capture certain patterns in the physical activity data (e.g. disengagement) which might require longer time series to unfold.

### Future research and conclusions

This study highlights that boundary conditions are central determinants of the phenomena observed, with direct implications for validating the conceptualization of physical activity maintenance. We see value in future research replicating and extending this work by testing alternative formulations of the boundary conditions examined here, as well as exploring additional boundary conditions (e.g. different populations, study designs). Such efforts would contribute to a broader understanding of how different operational definitions influence the observed patterns of behavior. Ultimately, this could support the development of a multidimensional map, where each dimension represents a boundary condition, that identifies combinations of boundary condition formulations under which the observed phenomena align with the target conceptual definition.

In parallel, we also see value in testing alternative conceptualizations of physical activity maintenance using the same approach. This would allow researchers to move toward consensus on which conceptual or operational elements should be retained or discarded, based on whether they are supported by empirical data patterns. Finally, findings emerging from this dialectic comparison of conceptualization and empirical phenomena can feed into the broader framework of epistemic iteration, providing a foundation for the theoretical refinement of behavior change models related to physical activity maintenance.

## Supplementary Material

Supplementary Materials.docx

## Data Availability

Statistical analyses were performed in R version 4.3.0. The data and R code used in the present study are available on OSF (https://osf.io/6kjwg/).

## Note

1 With the term phenomena we refer to empirical patterns that are detected from the data and serve as focal points for refining concepts and theories (Borsboom et al., 2021; Bringmann et al., 2022; Eronen & Bringmann, 2021).
