# Supplementary material for: Exploring boundary conditions of physical activity maintenance: A secondary analysis of time-series data from a weight-loss intervention
Source: Health Psychol Behav Med. 2025 Sep 11;13(1):2554980. doi: 10.1080/21642850.2025.2554980 (PMC12427485; doi:10.1080/21642850.2025.2554980)
Supplement: Supplementary Materials.docx [file RHPB_A_2554980_SM2108.docx]

Supplementary materials to the manuscript:

**Exploring boundary conditions of physical activity maintenance: A secondary analysis of time-series data from a weight-loss intervention.**

**Authors:**

Dario Baretta, Guillaume Chevance, Shadia J. Mansour-Assi, Victoria Lawhun Costello, David Wing, Eric B. Hekler, Jennifer Inauen, Job Godino & Claudio R. Nigg

**Table of Content**

[**Figure S1. Participant selection flow for the current study sample** 3](#_Toc201675580)

[**Table S1. Frequency of participants matching specific physical activity phenomena at the varying of the boundary conditions of timescale and threshold.** 4](#_Toc201675581)

[**Figure S2. Frequency of phase transitions across timescales and activtiy thresholds within the BL-Active group.** 5](#_Toc201675582)

[**Figure S3. Frequency of phase transitions across timescales and activtiy thresholds within the BL-Inactive group.** 6](#_Toc201675583)

[**Figure S4. Histograms and heatmaps showing phase transitions within the BL-Active group across 90% and 80% thresholds.** 7](#_Toc201675584)

[**Figure S5. Histograms and heatmaps showing phase transitions within the BL-Active group with the 150 MVPA threshold.** 8](#_Toc201675585)

[**Figure S6. Histograms and heatmaps showing phase transitions within the BL-Inactive group across 90% and 80% thresholds.** 9](#_Toc201675586)

**Figure S1. Participant selection flow for the current study sample**


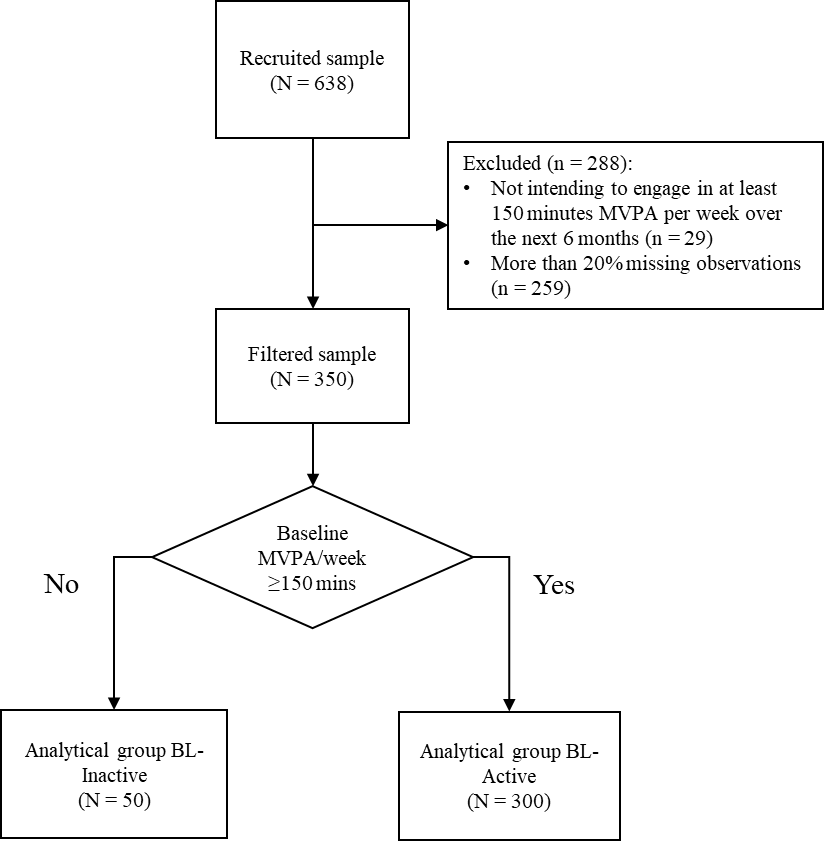


**Table S1. Frequency of participants matching specific physical activity phenomena at the varying of the boundary conditions of timescale and threshold.**

|  | **Time scale of change** | | | |
| --- | --- | --- | --- | --- |
|  | **7 days** | **14 days** | **28 days** | **56 days** |
| **BL-Inactive (n = 50)** |  |  |  |  |
| ***Target threshold (150 mins MVPA)*** |  |  |  |  |
| Below-threshold throughout (no transitions) | 2 (4%) | 6 (12%) | 9 (18%) | 13 (26%) |
| Reached and maintained | 3 (6%) | 5 (10%) | 14 (28%) | 20 (40%) |
| Reached and fluctuated | 45 (90%) | 39 (78%) | 27 (54%) | 17 (34%) |
| ***90% target threshold*** |  |  |  |  |
| Below-threshold throughout | 2 (4%) | 6 (12%) | 11 (22%) | 12 (24%) |
| Reached and maintained | 5 (10%) | 5 (10%) | 14 (28%) | 20 (40%) |
| Reached and fluctuated | 43 (86%) | 39 (78%) | 25 (50%) | 18 (36%) |
| ***80% target threshold*** |  |  |  |  |
| Below-threshold throughout | 2 (4%) | 4 (8%) | 8 (16%) | 13 (26%) |
| Reached and maintained | 5 (10%) | 7 (14%) | 14 (28%) | 20 (40%) |
| Reached and fluctuated | 43 (86%) | 39 (78%) | 28 (56%) | 17 (34%) |
| **BL-Active (n = 300)** |  |  |  |  |
| ***Target threshold (Baseline MVPA)*** |  |  |  |  |
| Threshold maintained throughout (no transitions) | 1 (0.3%) | 12 (4.0%) | 22 (7.3%) | 35 (11.7%) |
| Fell below and never returned | 30 (10.0%) | 70 (23.3%) | 126 (42.0%) | 187 (62.3%) |
| Fell below and fluctuated | 269 (89.7%) | 218 (72.7%) | 152 (50.7%) | 78 (26.0%) |
| ***90% target threshold*** |  |  |  |  |
| Threshold maintained throughout | 3 (1%) | 16 (5.3%) | 34 (11.3%) | 53 (17.7%) |
| Fell below and never returned | 18 (6%) | 55 (18.3%) | 108 (36.0%) | 167 (55.7%) |
| Fell below and fluctuated | 279 (93%) | 229 (76.3%) | 158 (52.7%) | 80 (27.7%) |
| ***80% target threshold*** |  |  |  |  |
| Threshold maintained throughout | 6 (2.0%) | 25 (8.3%) | 49 (16.3%) | 84 (28%) |
| Fell below and never returned | 11 (3.7%) | 49 (16.3%) | 88 (29.3%) | 138 (46%) |
| Fell below and fluctuated | 283 (94.3%) | 226 (75.3%) | 163 (54.3%) | 78 (26%) |
| ***150 mins MVPA*** |  |  |  |  |
| Threshold maintained throughout | 68 (22.7%) | 129 (43%) | 181 (60.3%) | 235 (77.7%) |
| Fell below and never returned | 12 (4.0%) | 30 (10%) | 42 (14.0%) | 42 (14.3%) |
| Fell below and fluctuated | 220 (73.3%) | 141 (47%) | 77 (25.7%) | 25 (8.0%) |

**Figure S2. Frequency of phase transitions across timescales and activity thresholds within the BL-Active group.**


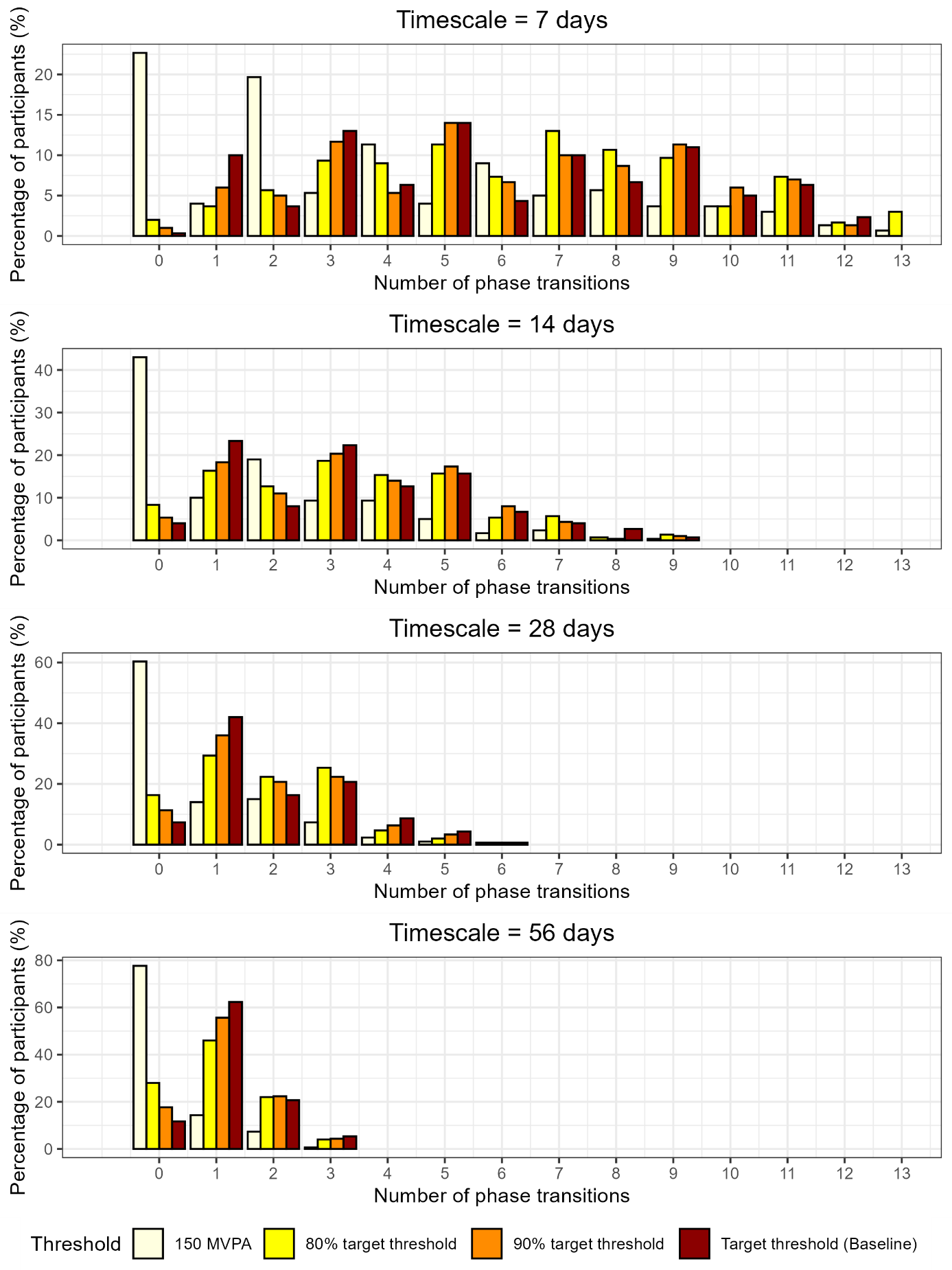


**Figure S3. Frequency of phase transitions across timescales and activity thresholds within the BL-Inactive group.**


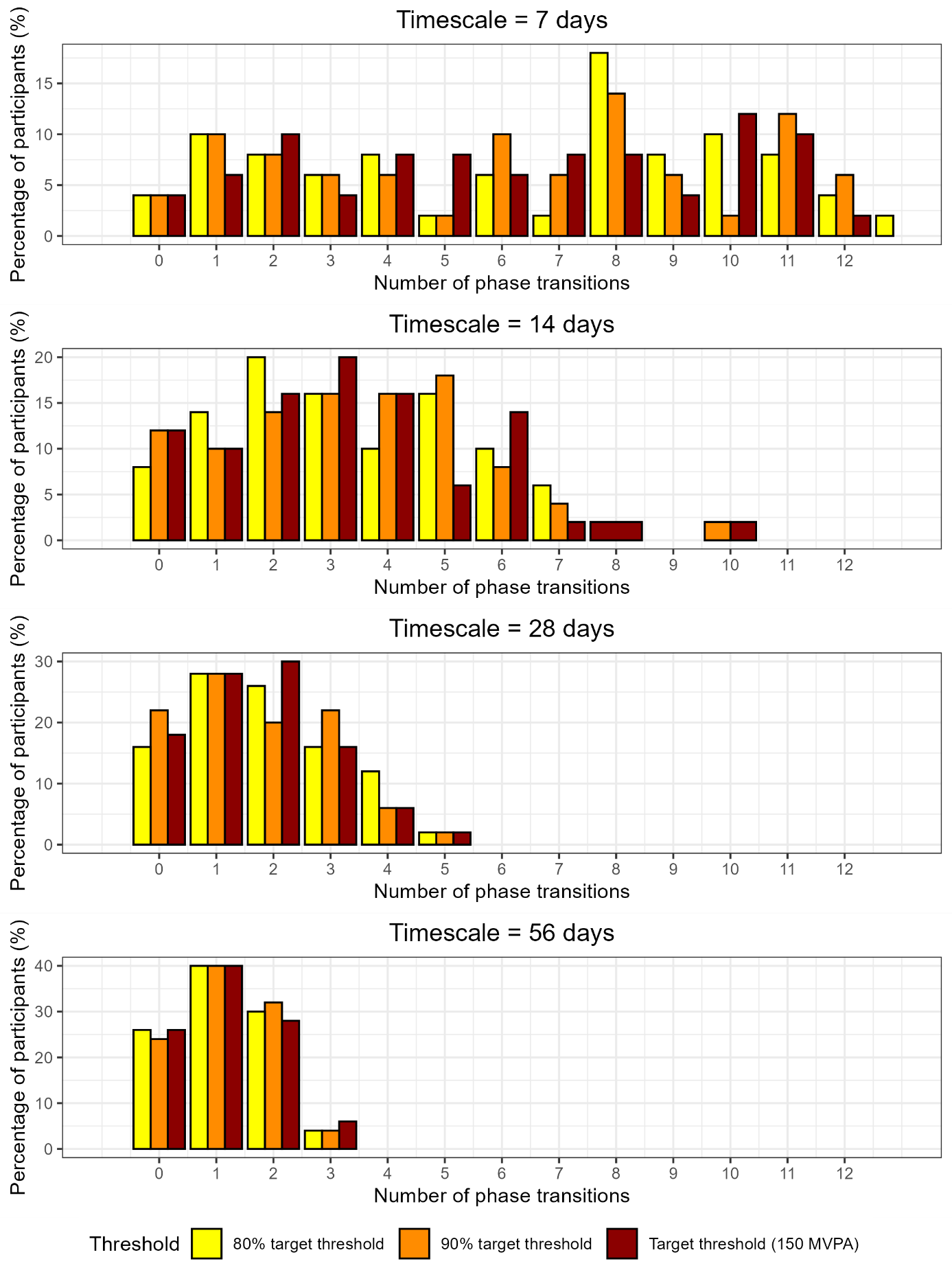


**Figure S4. Histograms and heatmaps showing phase transitions within the BL-Active group across 90% and 80% thresholds.**

**
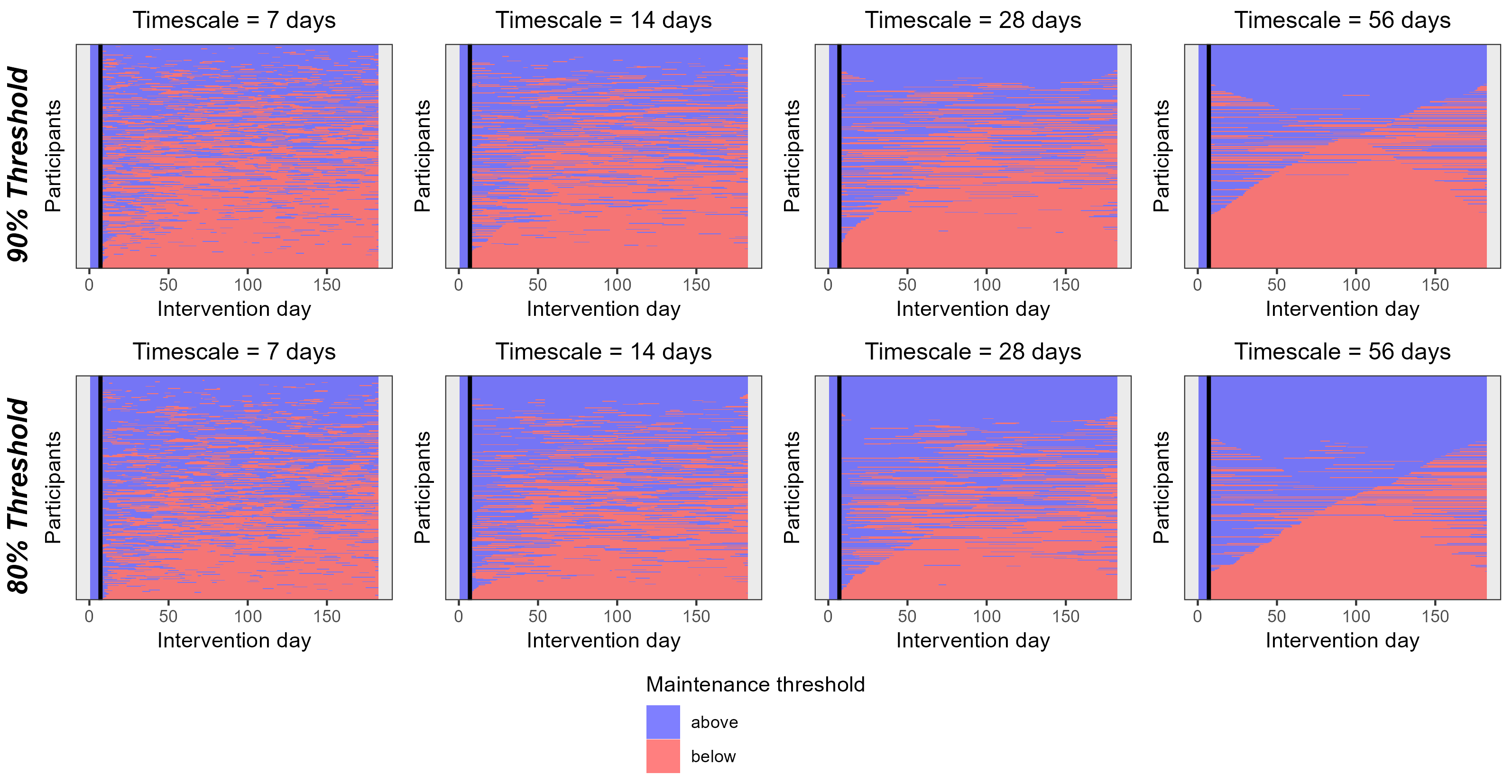
**

*Note.* The Y axis represents a unique participant, while x axis indicates progressive days since the intervention started. Vertical black lines indicate the first week of Fitbit data used to calculate baseline MVPA.

**Figure S5. Histograms and heatmaps showing phase transitions within the BL-Active group with the 150 MVPA threshold.**

**
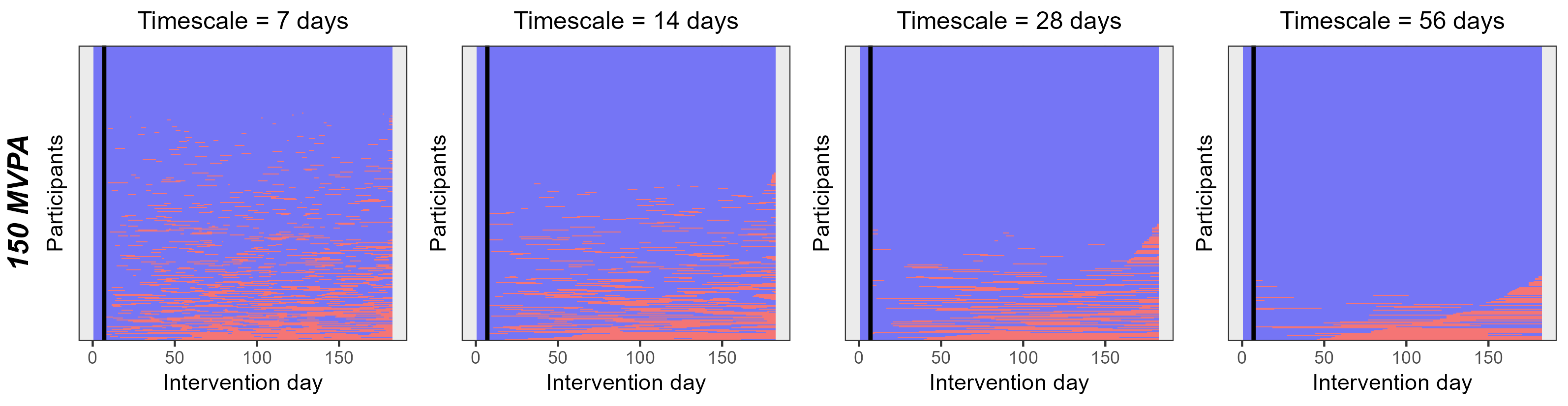
**

*Note.* The Y axis represents a unique participant, while x axis indicates progressive days since the intervention started. Vertical black lines indicate the first week of Fitbit data used to calculate baseline MVPA.

**Figure S6. Histograms and heatmaps showing phase transitions within the BL-Inactive group across 90% and 80% thresholds.**


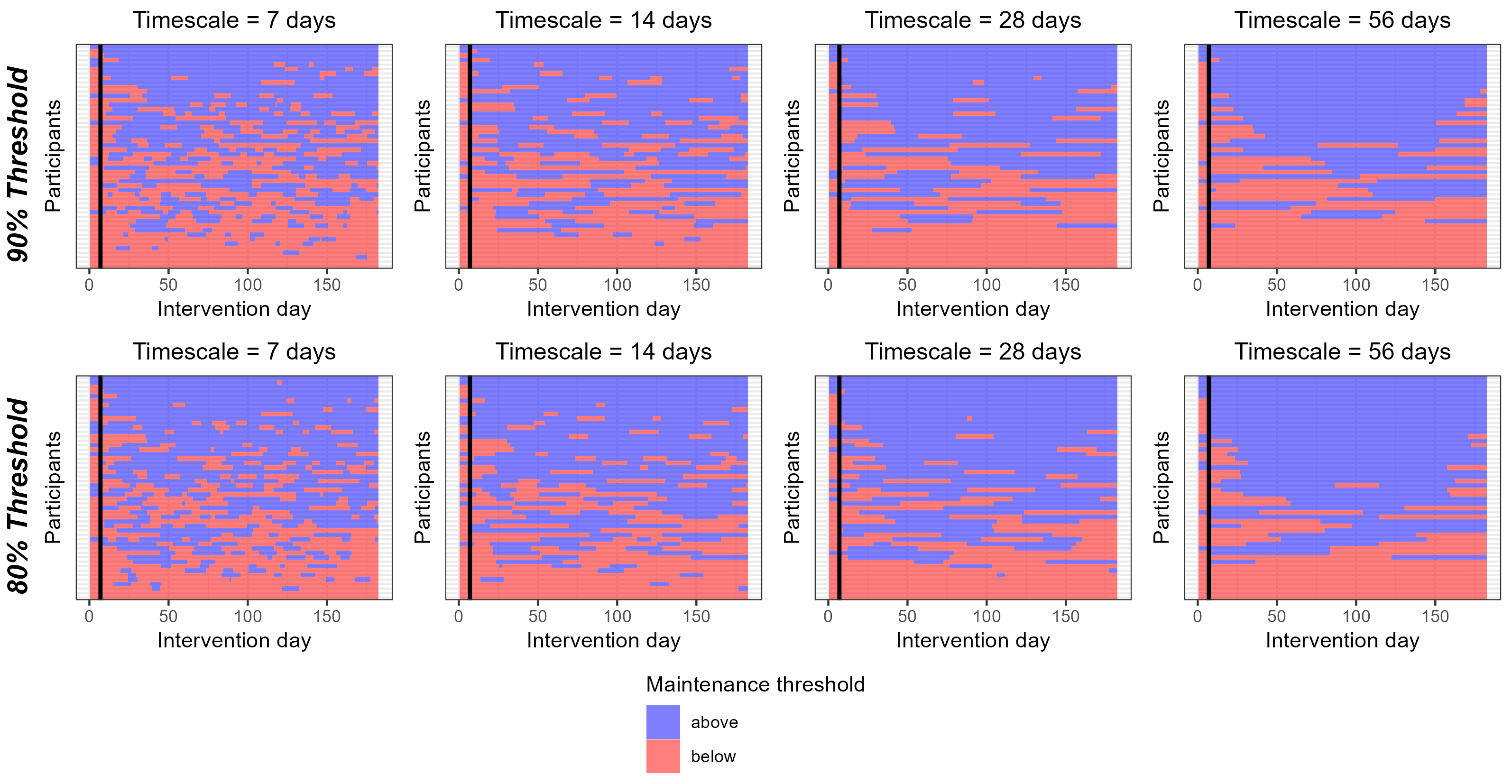


*Note.* The Y axis represents a unique participant, while x axis indicates progressive days since the intervention started. Vertical black lines indicate the first week of Fitbit data used to calculate baseline MVPA.
